# Extremophilic Yeasts as Next-Generation Eukaryotic Models: Mechanisms of Stress Integration, Systems Biology and Biotechnological Applications: A Review

**DOI:** 10.3390/jof12020092

**Published:** 2026-01-29

**Authors:** Francisco Padilla-Garfias, Antonio Peña

**Affiliations:** Departamento de Genética Molecular, Instituto de Fisiología Celular, Universidad Nacional Autónoma de México, Circuito Exterior s/n, Ciudad Universitaria, Mexico City 04510, Mexico

**Keywords:** extremophilic yeasts, eukaryotic model systems, integrated stress responses, systems biology, fungal biotechnology

## Abstract

Fungi, including yeasts, have played a central role in the development of knowledge about cell physiology and molecular biology as experimental eukaryotic models. However, much of this knowledge has been generated using classical organisms such as *Saccharomyces cerevisiae*, which display inherent limitations, as many cellular processes operate under extreme conditions, including high salinity, extreme pH, oxidative stress, exposure to toxic compounds, and temperature fluctuations. In this context, extremophilic and extremotolerant yeasts have emerged as complementary systems with strong potential for basic research and biotechnological applications. This review integrates recent advances in the taxonomic diversity, ecology, physiology, molecular mechanisms, and omics-based analyses of extremophilic yeasts, with a particular focus on how these organisms achieve stress integration through coordinated regulation of signaling pathways, metabolism, and organelle function. We discuss representative applications in environmental toxicology, bioremediation, and industrial bioprocesses, as well as their relevance in the context of climate change and space biotechnology. Finally, we outline key conceptual and methodological challenges and propose future perspectives that position extremophilic yeasts as next-generation eukaryotic models for investigating adaptation as a systems-level, constitutive cellular state under complex and dynamic stress conditions.

## 1. Introduction

Over the years, the study of fungi, particularly yeasts as unicellular eukaryotes, has profoundly shaped modern biological understanding [1]. Since the early twentieth century, yeasts have served as essential experimental tools because of their simplicity, ease of cultivation, and conservation of fundamental cellular processes relative to higher eukaryotes, enabling key discoveries in genetics, molecular biology, metabolism, and cellular physiology [1,2].

Although major advances have been achieved, fungal research has historically focused on a limited number of model species, mostly *Saccharomyces cerevisiae*. While this focus has been crucial for defining core principles of eukaryotic cell biology, it has also limited the environmental and physiological contexts in which these principles have been explored. In this sense, extremotolerant yeasts expand fungal biology by revealing adaptive cellular strategies that are largely inaccessible to classical model systems [3,4].

In recent decades, the expansion of research on nonconventional organisms has enabled the investigation of cellular resilience, metabolic plasticity, and the physiological limits of eukaryotic life, positioning extremophilic and extremotolerant yeasts as prominent experimental systems, as they reveal how eukaryotic cells integrate multiple environmental conditions into physiological states [5].

Extremophilic organisms require extreme conditions for optimal growth, whereas extremotolerant organisms can survive under such conditions but grow optimally outside them [6]. Importantly, the occurrence of a yeast in an extreme environment is not sufficient to establish extremophily, which must be supported by reproducible isolation and physiological validation demonstrating optimal growth under the relevant extreme conditions [5,6].

Current challenges, including contamination by toxic compounds, increasing industrial demands, and climate change, have intensified interest in the metabolic and physiological capabilities of extremophilic and extremotolerant yeasts.

### 1.1. Relevance of Fungi and Yeasts as Biological Models

The relevance of fungi in cellular biology and physiology derives from their structural simplicity, experimental tractability, and the conservation of fundamental cellular processes of eukaryotes [1,7]. Using yeasts, fundamental mechanisms of genome maintenance, gene expression, cell cycle regulation, organelle biogenesis, intracellular trafficking, and energy metabolism have been elucidated, largely through studies conducted in *S. cerevisiae* and *Schizosaccharomyces pombe* established general principles later validated in animal and human cells, showing functional conservation across eukaryotes [7,8].

Yeasts have enabled the development of methodologies foundational to biomedical and biotechnological research, facilitating the analysis of global regulatory networks and the integration of cellular signaling, metabolism, and stress responses [2,7,8]. Studies in fungi have been central to understanding how eukaryotic cells adapt to environmental challenges, including oxidative, osmotic, thermal, and nutritional stress, revealing core mechanisms of redox homeostasis, mitogen-activated protein kinase (MAPK) signaling, environment-dependent gene expression, and metabolic remodeling. These findings have also provided insight into pathological processes linked to oxidative stress, such as aging, cancer, and neurodegenerative diseases [9,10,11].

In industrial biotechnology and environmental research, yeasts are widely used as experimental and applied platforms, supporting studies on fermentation, metabolite production, recombinant protein expression, and tolerance to extreme conditions and contaminants [3,4,12].

### 1.2. Limitations of Classical Models and Need for Novel Fungal Systems

Despite its extensive experimental use, *S. cerevisiae* exhibits clear physiological limitations, since its tolerance to salinity, extreme pH, radiation, desiccation, and high concentrations of metals and xenobiotics is comparatively low, and its metabolism is optimized for simple carbon sources such as glucose [10,12]. These features constrain its suitability for studying cellular responses and degradation processes under chemically and physically demanding conditions [2].

These limitations are significant because many natural environments are polyextreme, combining multiple stress factors simultaneously, underscoring the need for alternative fungal models, such as nonconventional yeasts, that better reflect the conditions under which biological systems persist [6,13].

### 1.3. Emergence of Extremophilic Yeast

Extremophily, defined as the capacity to grow or survive under physicochemical conditions lethal to most organisms, is widespread across all domains of life. In fungi, and particularly yeasts, extremophily relies on diverse adaptive strategies that enable persistence under a broad range of environmental constraints, encompassing osmotic, chemical, physical, and radiative stresses [6,13,14].

**Figure 1:**
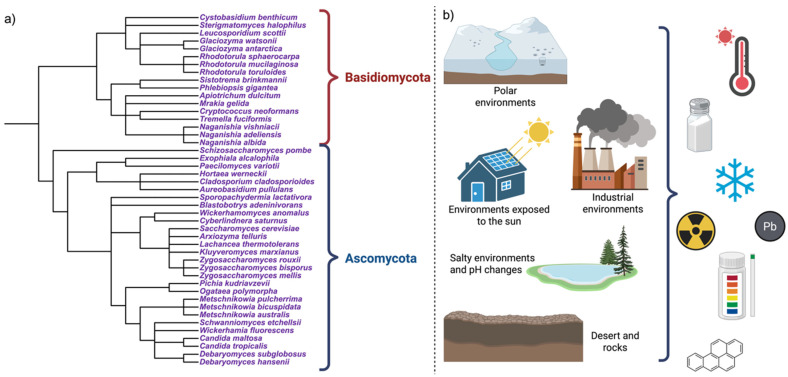
Diversity and ecological distribution of extremophilic yeasts. (**a**) A simplified phylogenetic overview highlights the convergent emergence of extremophilic traits across Ascomycota and Basidiomycota, with multiple stress tolerances arising independently during evolution. (**b**) Extremophilic yeasts inhabit polyextreme environments, where multiple physical, chemical, and energetic stressors co-occur, including high salinity, extreme pH, radiation, desiccation, metals, and xenobiotics. Created in BioRender. Padilla, F. (2026) https://BioRender.com/vreslc0.

These yeasts include halotolerant species (*Debaryomyces hansenii*, *Hortaea werneckii*), psychrotolerant taxa (*Mrakia*, *Naganishia*), acidophilic yeasts (*Aureobasidium pullulans*, *Candida* spp.), radiotolerant organisms (melanized and pigmented yeasts), metal-tolerant species (*Rhodotorula*, *Yarrowia*), as well as polyextremophilic organisms [13,15], as can be seen in Figure 1.

This review addresses this knowledge gap by integrating ecological, physiological, and molecular perspectives into a unified framework for interpreting extremophilic yeast biology. We argue that extremophilic yeasts should not be viewed merely as more tolerant versions of classical model organisms, but as systems in which stress integration represents a defining organizational principle of cellular biology. Whereas in *S. cerevisiae* stress responses are typically inducible and transient, reflecting adaptation to discrete perturbations under controlled conditions, extremophilic yeasts are chronically exposed to multiple, overlapping environmental stresses. As a consequence, many of their adaptive features are constitutively embedded within basal cellular organization rather than deployed as short-term responses. Viewed through this lens, extremophilic yeasts emerge as next-generation eukaryotic models for dissecting adaptation to complex and persistent stress conditions.

## 2. Extremophilic Yeasts: Diversity, Physiology and Ecological Niches

Extremophilic yeasts are a diverse group of eukaryotes adapted to physical, chemical, and energetic stresses that exceed the tolerance limits of most biological systems, and thus offer complementary platforms to classical model yeasts, typically optimized for stable, nutrient-rich conditions, for probing the functional boundaries of the eukaryotic cell [13,16]. Crucially, yeast extremophily is best viewed as a dynamic continuum, spanning moderate extremotolerance to polyextremophily and mirrored by broad taxonomic distribution and diverse ecological niches [6,15].

Extreme habitats are characterized by combinations of stressors that compromise microbial life, with nutrient limitation frequently acting as an additional constraint [17,18]. These conditions occur not only in natural systems but also in artificial and anthropogenic environments, including fermented foods, where low pH, high osmolarity, ethanol, and oxygen limitation co-occur, as well as in acid mine drainages and acidic rivers (e.g., Río Tinto, Spain), and exposed, nutrient-poor surfaces colonized by black fungi and melanized yeasts (Figure 1) [6,19,20,21].

### 2.1. Taxonomic Diversity and Evolutionary Implications

Extremophilic yeasts are distributed across major fungal lineages, including Ascomycota, Basidiomycota, and melanized black fungi, supporting the view that extremophily has emerged convergently during fungal evolution [22]. This distribution indicates that tolerance to extreme conditions relies on conserved eukaryotic features that are differentially deployed according to the ecological context (Figure 1). Representative taxa are summarized in Table 1 (adapted and updated from Buzzini et al., 2018) [13].

Several yeasts display polyextremophilic traits, with representative examples summarized in Table 2 (adapted and updated from Buzzini et al., 2018) [13].

Within Ascomycota, genera such as *Debaryomyces*, *Yarrowia*, *Candida*, and *Aureobasidium* are notable for their metabolic and physiological versatility. In contrast, basidiomycetous yeasts, including pigmented genera such as *Rhodotorula*, *Naganishia*, and *Vishniacozyma*, are frequently associated with tolerance to oxidative stress, radiation, and cold environments. Melanized (black) yeasts (*Hortaea*, *Exophiala*, *Knufia*) combine slow growth with exceptional resistance to multiple stresses [13,16].

From an evolutionary perspective, selection in extreme environments, typically polyextreme, favors genomic plasticity, gene redundancy, and robust regulatory architectures that support metabolic flexibility and long-term survival. This configuration contrasts with *S. cerevisiae*, which is optimized for rapid proliferation in nutrient-rich, competitive settings rather than its persistence under extreme conditions [15,23].

### 2.2. Mechanisms of Extremotolerance

**Figure 2:**
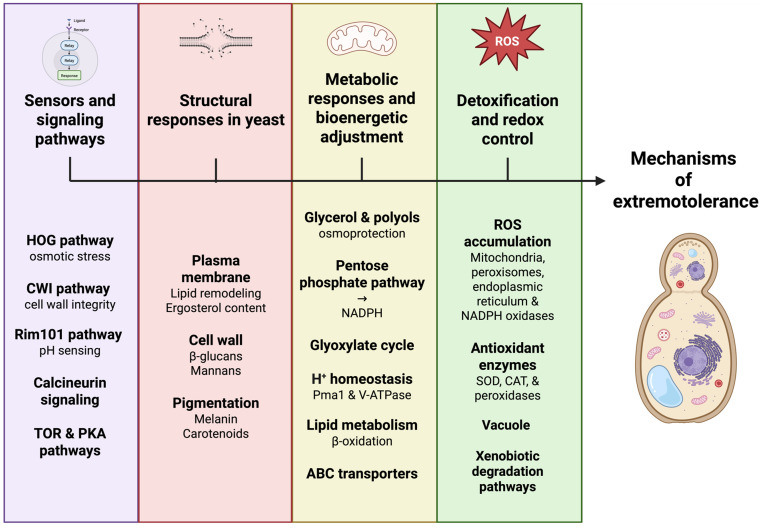
Integrated cellular stress-response networks in extremophilic yeasts. Extremophilic yeasts withstand harsh environments through the coordinated integration of multiple cellular stress-response networks rather than by isolated adaptive pathways. Environmental signals are sensed by interconnected signaling cascades, including HOG, CWI, Rim101, calcineurin, TOR, and PKA pathways, which dynamically regulate structural, metabolic, and redox responses. Adaptive remodeling of the plasma membrane, cell wall, and protective pigmentation enhances cellular robustness, while metabolic reprogramming supports energy balance and redox homeostasis. Detoxification systems, antioxidant defenses, and vacuolar transport collectively maintain intracellular stability. Together, these interlinked processes generate emergent stress tolerance as a systems-level property. Created in BioRender. Padilla, F. (2026) https://BioRender.com/suie93g.

Tolerance to extreme conditions in yeasts arises from the coordinated integration of signaling, regulatory, metabolic, and structural responses that together sustain cellular functionality under stress [24]. The following subsections summarize the major functional groups of extremophilic yeasts and the core mechanisms supporting their adaptation (Figure 2).

#### 2.2.1. Halotolerant and Halophilic Yeasts

Halotolerant and halophilic yeasts grow in high-salinity environments such as solar salterns, hypersaline lakes, industrial brines, fermented foods, and extreme marine ecosystems [25]. These habitats impose combined stresses driven by elevated osmotic pressure, reduced water activity (a_w_), and ionic toxicity primarily associated with Na^+^ and Cl^−^ [26,27,28].

Among the most studied halotolerant yeasts, *D. hansenii* and *H. werneckii* display growth across exceptionally broad NaCl ranges, tolerating concentrations approaching 20–23% [29,30].

Under high salinity, halotolerant yeasts face combined osmotic and ionic stress that is counteracted through regulated osmotic homeostasis systems, prominently the High Osmolarity Glycerol (HOG) pathway, which integrates osmotic signals with transcriptional, metabolic, and structural responses [27,28]. Consistent with adaptation to chronic salinity, osmoprotection is frequently implemented through species-specific solute strategies: in *D. hansenii*, glycerol accumulation and transport are supported by increased copy number of the glycerol transporter STL1, whereas Y. *lipolytica* relies primarily on erythritol accumulation [31,32].

*D. hansenii*, *H. werneckii*, and *A. pullulans*, under high salinity, remodel plasma membrane lipid composition by adjusting unsaturated fatty acid content and ergosterol levels, thereby preserving membrane fluidity and integrity. Importantly, lipid remodeling is both species- and solute-dependent: in *Z. rouxii*, responses differ when osmotic stress is imposed by salt versus high sugar concentrations [33,34,35,36,37,38].

Cell wall integrity under high salinity is maintained through activation of the CWI (Cell Wall Integrity) pathway and its functional coordination with HOG signaling. This crosstalk promotes cell surface reinforcement, exemplified by *FLO11* overexpression–associated salt tolerance in *Z. rouxii* and by Na^+^-induced phosphorylation of cell wall biosynthetic enzymes reported in *D. hansenii* [39,40,41,42].

Ionic homeostasis is a core component of halotolerance and is mediated by regulated Na^+^ and K^+^ transport systems, including TRK, ENA, and TOK families. In *H. werneckii*, expansion of ion transport–related genes may increase regulatory flexibility under fluctuating salinity, yet comparative analyses indicate that transporter abundance alone does not predict salt tolerance. Instead, in *D. hansenii*, high-salinity resistance reflects coordinated integration of ionic transport with osmotic, metabolic, and structural adaptation layers [41,43,44,45].

In some halotolerant yeasts, pigmentation provides an additional, but not universal, layer of protection. In *H. werneckii,* cell wall-associated melanin contributes to resistance against oxidative stress and radiation in hypersaline settings, whereas carotenoids in yeasts such as *Rhodotorula mucilaginosa* can modulate sensitivity to salt stress, consistent with a membrane-protective antioxidant role [13,45,46].

#### 2.2.2. Osmotolerant and Osmophilic Yeasts

Osmotolerant and osmophilic yeasts maintain viability and metabolic activity under low a_w_ conditions imposed by high solute concentrations, particularly sugars (typically ≥50–60% *w*/*w*; a_w_ ≤ 0.85) [47]. Such environments generate chronic hyperosmotic stress that perturbs membrane properties, protein stability, and cytosolic organization, thereby requiring integrated physiological strategies to sustain cellular homeostasis during prolonged exposure [27,48,49]. Ecologically, these yeasts are predominantly associated with sugar-rich substrates and fermentative environments characterized by extreme osmotic pressure and limited a_w_ [49]. Notably, osmophily reflects adaptation to sustained low-aw conditions rather than salt tolerance alone, as growth of the conventional model yeast *S. cerevisiae* under extreme sugar concentrations despite its ability to tolerate ionic osmotic stress (e.g., ~1 M KCl). Representative taxa include *Zygosaccharomyces* species, particularly *Z. rouxii* and *Z. mellis*, which proliferate under extreme sugar concentrations, as well as *Candida* species such as *Candida zemplinina* and *Candida versatilis* recurrently detected in osmotically demanding fermentations [48,50,51].

Physiological adaptation to extreme osmotic conditions in osmotolerant and osmophilic yeasts relies primarily on the accumulation of compatible solutes, with glycerol being the dominant osmoprotectant [27,52]. Effective osmoprotection depends not only on glycerol synthesis but also on its retention, which is achieved through tight regulation of glycerol flux across the plasma membrane, particularly via control of the Fps1 channel, which limits osmolyte efflux under hyperosmotic conditions [47]. In highly tolerant species such as *Z. rouxii*, HOG activation coordinates and regulates glycerol metabolism [27,53].

In addition to osmotic stress, high sugar concentrations impose secondary chemical stress in osmophilic yeasts, affecting protein stability, membrane properties, and redox balance [27]. These effects are mitigated through adaptive membrane remodeling; however, studies in *Z. rouxii* demonstrate that physiological responses differ, depending on whether osmotic stress is imposed by sugars or salts, highlighting solute-specific regulation [47,52,53].

In osmophilic yeasts, the cell wall functions as a mechanical buffer against turgor changes under hyperosmotic conditions, with CWI pathway activation reinforcing cellular stability during prolonged exposure to low a_w_ [47,52].

Metabolically, osmophilic yeasts undergo efficiency-oriented reprogramming characterized by slow but sustained growth, prioritizing cellular homeostasis over rapid proliferation under chronic osmotic stress [28,52]. This configuration supports long-term viability in environments with persistently low water availability [48].

#### 2.2.3. Piezotolerant and Piezophilic Yeasts

Piezophilic and piezotolerant yeasts are specialized unicellular fungi capable of sustaining metabolic activity under high hydrostatic pressure, typically encountered in deep marine environments where pressures can exceed 10–100 MPa. Unlike most environmental stressors, hydrostatic pressure acts directly on molecular interactions, perturbing protein conformation and lipid bilayer organization, with system-wide consequences for cellular stability and function [54,55].

Ecologically, piezophilic and piezotolerant yeasts are primarily isolated from deep-sea sediments, abyssal waters, and hadal environments, where high hydrostatic pressure coexists with low temperature, limited nutrients, and frequent hypoxia [56]. Several genera, including *Rhodotorula*, *Cryptococcus*, *Candida*, and *Debaryomyces*, include species capable of growth or survival at pressures far exceeding those tolerated by mesophilic yeasts, reflecting adaptation to complex deep-ocean niches [54].

At the cellular level, high hydrostatic pressure destabilizes the plasma membrane by reducing bilayer fluidity, altering permeability, and impairing membrane protein function. Piezotolerant yeasts counteract these effects through lipid remodeling, including increased unsaturated fatty acids and adjusted sterol content [57].

High hydrostatic pressure compromises protein stability by disrupting protein complexes and enzyme conformation, constraining central metabolic pathways. Piezotolerant yeasts mitigate these effects through enhanced reliance on molecular chaperone systems and selective regulation of key metabolic enzymes, preserving catalytic efficiency under extreme physical conditions [55,58].

At the metabolic level, high hydrostatic pressure drives reprogramming of central carbon metabolism toward energy-efficient configurations associated with reduced growth. In piezotolerant yeasts, pressure alters the balance between respiratory and fermentative metabolism and impacts mitochondrial function, collectively favoring long-term survival under chronic pressure stress [56].

Adaptation to high hydrostatic pressure also involves indirect effects on redox homeostasis. Although pressure does not directly generate reactive oxygen species (ROS), pressure-induced perturbations of mitochondrial function can disrupt redox balance, prompting reinforcement of antioxidant and detoxification systems to maintain cellular and metabolic stability [57,59].

#### 2.2.4. Thermotolerant Yeasts

Thermotolerant yeasts sustain growth and metabolic activity at temperatures exceeding those tolerated by most mesophilic species, typically above 40–45 °C and, in some cases, approaching 50–52 °C, while the upper thermal boundary for eukaryotic life rarely exceeds 60–62 °C [60].

Among thermotolerant yeasts, *Kluyveromyces marxianus* sustains growth between 40 and 50 °C with a high metabolic activity, while related species such as *Ogataea polymorpha* and *Ogataea thermomethanolica* grow at temperatures approaching 48–50 °C, supporting their use as models of thermal stress physiology and industrial biotechnology [61,62]. *Lachancea thermotolerans* exhibits moderate thermotolerance (≈37–40 °C), whereas yeasts associated with homeothermic hosts, including *Arxiozyma telluris* and *Cyniclomyces* spp., display elevated minimum growth temperatures and limited cold tolerance, consistent with host-associated thermal adaptation [63,64].

Thermotolerance in yeasts is primarily governed by reinforced proteostasis, including sustained activity of molecular chaperones such as Hsp70, Hsp90, and Hsp104, which limit protein misfolding and aggregation under prolonged thermal stress [60,65].

Thermal adaptation in yeasts involves remodeling of membrane composition, particularly sphingolipids and ergosterol, to sustain membrane function at elevated temperatures. Consistently, thermotolerant species display altered regulation of sterol biosynthesis genes such as *ERG3*, *ERG4*, and *ERG5* [66,67].

Thermotolerant yeasts further accumulate compatible solutes such as trehalose and glycerol that stabilize proteins and membranes under heat stress, while increased expression of ABC (ATP-binding cassette) transporters involved in lipid homeostasis, notably PDR18, supports plasma membrane integrity [68,69]. These responses are embedded within broader regulatory frameworks involving transcription factors such as Hsf1 and Msn2/4, together with genome organization and epigenetic regulation [70,71].

#### 2.2.5. Psychrophilic and Psychrotolerant Yeasts

Psychrophilic and psychrotolerant yeasts are adapted to cold environments that impose strong constraints on biological activity, including low temperatures near or below 0 °C, nutrient limitation, elevated radiation exposure, and restricted liquid water availability [72].

Cold-adapted yeasts have emerged convergently across multiple evolutionary lineages, with a strong predominance of Basidiomycota [73,74,75]. Genera such as *Mrakia*, *Naganishia*, and *Vishniacozyma* are frequently isolated from glaciers, permafrost, ice cores, and Antarctic environments, while *Cryptococcus*, *Rhodotorula*, *Leucosporidium*, and *Cystobasidium* further expand the ecological and functional diversity of psychrotolerant yeasts [72].

Cold adaptation in psychrophilic yeasts relies on plasma membrane remodeling and sustain transport and signaling at low temperatures, under transcriptional control [76,77]. These yeasts produce cold-active enzymes with enhanced conformational flexibility, enabling efficient catalysis near or below freezing, and synthesize antifreeze proteins and compatible solutes such as glycerol and trehalose that limit ice formation and stabilize macromolecules, as shown in genomic and physiological studies of *Glaciozyma antarctica* [78,79,80,81].

Psychrophilic yeasts exhibit slow but efficient growth strategies that prioritize long-term survival and cellular maintenance over rapid proliferation, supported by coordinated genomic and transcriptomic reprogramming involving protein quality control and stress-related responses in chronically cold environments [79].

The capacity of psychrophilic yeasts to sustain growth and catalytic activity at low temperatures underpins their application in cold fermentations, production of cold-active enzymes, and bioremediation processes in cold environments [81,82].

#### 2.2.6. Acidophilic and Alkalitolerant Yeasts

Yeasts capable of growth under extreme acidic or alkaline pH illustrate how eukaryotic cells sustain metabolism when electrochemical gradients and macromolecular stability are severely challenged [83,84]. Acidic environments (pH < 3) impose cytosolic acidification and oxidative stress, whereas alkaline conditions (pH > 9) restrict proton-motive force generation and H^+^-coupled transport, requiring coordinated control of intracellular pH and metabolic regulation in both cases [20,85,86].

In yeasts adapted to extreme pH, intracellular pH homeostasis relies on coordinated activity of the plasma membrane H^+^-ATPase (Pma1) and the vacuolar H^+^-ATPase (V-ATPase), which together mediate proton extrusion and compartmental sequestration. The efficiency of these systems depends on limiting passive proton fluxes, thereby buffering the impact of extreme external pH on cellular bioenergetics [83,84,85].

Acidophilic and acidotolerant yeasts typically integrate pH regulation with detoxification and oxidative stress control. *A. pullulans* exemplifies pronounced physiological plasticity, growing across a broad pH range (≈2.1–6.5) while tolerating elevated metal concentrations [13,87]. Similarly, yeasts isolated from acid mine drainage environments combine metal resistance, oxidative stress management, and substrate versatility under oligotrophic conditions [20]. In fermentative settings, *Pichia kudriavzevii* and *Zygosaccharomyces bailii* tolerate low pH in combination with ethanol, preservatives, or elevated temperature, reflecting integrated stress-response architectures rather than pH adaptation alone [84,85,86].

Basidiomycetous yeasts also colonize acidic environments, sustaining metabolic activity under chemical stress. Species such as *Apiotrichum dulcitum* and *Leucosporidium scottii* display bioremediation potential through degradation of aromatic compounds under conditions unfavorable for most eukaryotes [86]. *Cryptococcus* tolerates acidity and heavy metals through integrated pH control and detoxification responses, while *R. mucilaginosa* further illustrates this strategy via metabolic plasticity and carotenoid production supporting function under combined acidity and contaminant stress [20,87].

In alkaline environments, limited proton availability challenges cellular bioenergetics and increases the demand for ionic homeostasis. Most yeasts are alkalitolerant rather than strictly alkaliphilic, growing optimally near neutrality while tolerating elevated pH [86]. Adaptation relies on sustained Pma1 activity, Na^+^/H^+^ antiporters such as Nha1, and K^+^ uptake systems, coordinated by the pH-responsive Rim101 pathway [85,87]. In parallel, metabolic reprogramming toward the production of acidic by-products contributes to local microenvironment acidification, partially compensating for external proton limitation. These responses are integrated through regulatory nodes shared with other stress pathways, including Hog1p, calcineurin, target of rapamycin (TOR), protein kinase A (PKA), and Msn2/4-type regulators [85].

*Wickerhamomyces anomalus* exhibits exceptional pH tolerance, growing from near pH 2 to above pH 12, reflecting robust intracellular pH control networks [13]. *A. pullulans* similarly tolerates alkaline conditions up to approximately pH 10.5 [88]. However, extreme pH tolerance alone does not necessarily translate into experimental suitability. Several basidiomycetous yeasts reach high alkaline pH limits, yet their genetic manipulation remains limited. In contrast, ascomycetous species such as *W. anomalus* offer a favorable balance between broad pH tolerance and experimental tractability, making them practical models for mechanistic studies of pH adaptation [13].

#### 2.2.7. Radiation- and Desiccation-Resistant Yeasts

Radiation- and desiccation-resistant yeasts represent a distinctive group of extremotolerant eukaryotes adapted to environments characterized by chronic exposure to ultraviolet (UV) or ionizing radiation and severe water limitation, such as polar deserts, high-altitude ecosystems, exposed rock surfaces, and hyper-arid habitats. These stresses generate largely overlapping cellular damage profiles, including oxidative stress, DNA lesions, lipid and protein oxidation, and loss of membrane integrity, explaining why resistance to radiation and desiccation frequently co-occur and rely on convergent protective strategies [13,89,90]. For this reason, although radiation and desiccation are distinct environmental pressures, they trigger largely similar cellular responses in yeasts and are therefore discussed together in this section for clarity and coherence.

Resistance to radiation and desiccation has emerged convergently across multiple fungal lineages, including basidiomycetous yeasts such as *Rhodotorula*, *Naganishia*, *Papiliotrema*, and *Vishniacozyma*, as well as ascomycetes and melanized “black fungi” such as *Aureobasidium*, *Hortaea*, *Knufia*, and *Exophiala* [91]. A defining feature of many of these organisms is pigmentation, which plays a central structural and protective role. Cell wall–associated melanin acts as a physicochemical barrier that absorbs radiation, limits oxidative processes, and contributes to ROS neutralization, as documented in melanized yeasts such as *H. werneckii*, *Cryptococcus neoformans*, and *A. pullulans* [92]. In carotenoid-producing yeasts such as *Rhodotorula* spp., lipophilic pigments reduce membrane peroxidation and oxidative damage, reinforcing tolerance to combined radiation and desiccation stress [93].

Efficient protection and repair of genetic material constitute another core component of resistance. Radiation- and desiccation-tolerant yeasts display robust DNA repair capacity, involving homologous recombination together with base excision repair (BER) and nucleotide excision repair (NER) pathways that remove oxidative and radiation-induced lesions In addition to repair efficiency, maintenance of nuclear organization and the ability to re-establish cell cycle progression after damage are critical for survival under repeated stress exposure [94].

Desiccation imposes severe constraints on macromolecular stability due to the loss of water as a solvent. To counteract this, resistant yeasts accumulate compatible solutes such as trehalose and polyols, together with molecular chaperones and intrinsically disordered proteins that stabilize proteins and membranes during dehydration and enable recovery upon rehydration These mechanisms support entry into a metabolically quiescent yet structurally preserved state, favoring persistence rather than rapid growth [72,90].

Cellular architecture and energy economy are central to resistance, in melanized yeasts such as *Knufia* and *Exophiala*, compact growth, envelope robustness, and investment in pigmentation and cellular maintenance support this strategy. At the network level, resistance integrates oxidative stress defenses (enzymes such as superoxide dismutase (SOD), catalase (CAT), and peroxidases), DNA repair pathways, membrane and cell wall remodeling, and quiescence programs that preserve viability during dehydration [95,96].

Owing to this combination of traits, radiation- and desiccation-resistant yeasts have been proposed as valuable eukaryotic models to probe the functional limits of life and polyextremotolerance, particularly in astrobiological contexts. Species such as *Aureobasidium* and the cryoresistant fungus *Cryomyces* have been included in space agency-associated experiments involving exposure to radiation, vacuum, microgravity, and thermal cycling, providing insights into cellular resilience beyond typical terrestrial ranges [95,97,98].

#### 2.2.8. Metalotolerant Yeasts

Metal-tolerant yeasts confront one of the most persistent abiotic stresses, as heavy metals do not degrade and exert toxicity through convergent mechanisms involving redox imbalance, disruption of essential cofactors, and macromolecular damage [99]. Such conditions characterize both natural and anthropogenic environments, including acid mine drainages, contaminated soils and waters, and metal-impacted marine sediments [100].

Metal tolerance is widely distributed among Ascomycota and Basidiomycota and often forms part of a broader polyextremotolerant phenotype integrated with tolerance to salinity, extreme pH, radiation, or desiccation [101]. Rather than relying on a single mechanism, metal-tolerant yeasts deploy coordinated strategies encompassing exclusion, detoxification, sequestration, and metabolic adaptation. Representative examples include *Candida* species and *R. mucilaginosa*, which tolerate metals such as Cr(VI), Cd, Cu, and Ni, as well as *Y. lipolytica* and *D. hansenii*, which combine metal tolerance with biomineralization capacity or halotolerance [101,102,103].

At the cellular level, metal resistance in yeasts emerges from a coordinated set of defense mechanisms that limit metal ion entry, promote active extrusion, and mitigate intracellular toxicity. Regulated transport systems, including P-type ATPases and ABC transporters, restrict metal accumulation by dynamically modulating uptake pathways for essential metals such as Fe, Cu, and Mn, thereby minimizing nonspecific incorporation of toxic ions, while efficient efflux underpins multi-metal tolerance in *Candida* species [104,105,106,107,108]. Metals that enter the cell are detoxified through intracellular sequestration, primarily via chelation by glutathione and phytochelatins, which immobilize ions such as Cd and Pb and facilitate their transport to the vacuole through ABC transporters, a central detoxification route in *Candida* spp. [109]. Cysteine-rich metallothioneins further contribute to metal binding and buffering, supporting intracellular metal homeostasis [110,111,112]. In parallel, some yeasts actively transform metals into less toxic or exploitable forms, including the reduction in Cr(VI) to Cr(III) in *Candida* species and biomineralization processes in *Y. lipolytica*, linking detoxification with elemental transformation and resource recovery [101,110]. At the cell surface, the cell wall and extracellular matrix provide an additional protective barrier through biosorption mediated by chitin, glucans, mannans, and exopolysaccharides, a mechanism well documented in *Rhodotorula* spp. and widely exploited in biosorption-based remediation strategies [101,113]. These defenses are complemented by antioxidant systems that limit secondary macromolecular damage, with carotenoid production in pigmented yeasts such as *R. mucilaginosa* providing an additional layer of non-enzymatic protection that reinforces multi-metal tolerance [114].

Collectively, metalotolerance in yeasts emerges from the integration of ionic control, redox homeostasis, vacuolar function, and metabolic remodeling rather than from isolated detoxification pathways. This systems-level organization underlies both their persistence in contaminated environments and their applicability as eukaryotic models for studying adaptation under chronic chemical stress [101,113].

#### 2.2.9. Polycyclic Aromatic Hydrocarbon and Xenobiotic Degrading Yeasts

Yeasts tolerant to polycyclic aromatic hydrocarbons (PAHs) are exposed to chemical stress imposed by the hydrophobicity, chemical stability, and intrinsic toxicity of these compounds, which readily partition into membranes and lipid-rich compartments [115]. PAH exposure disrupts membrane integrity and generates reactive intermediates during biotransformation, intensifying oxidative stress and imposing a substantial redox burden [116]. Consequently, survival under PAH stress requires tight integration of detoxification pathways, redox control, and metabolic reorganization [117].

PAH tolerance extends beyond substrate utilization, as many yeasts degrade these compounds cometabolically while simultaneously coping with their toxic effects. This capacity frequently coexists with tolerance to additional stresses, including high salinity, nutrient limitation, and heavy metals, situating PAH-tolerant yeasts within a broader polyextremotolerant framework [116,118].

Adaptation to PAHs is driven by the xenome, comprising enzymatic systems responsible for xenobiotic biotransformation. Initial activation of PAHs is mediated by cytochrome P450 monooxygenases (CYP), followed by epoxide hydrolases (EH), a process that facilitates degradation but generates electrophilic intermediates and elevates intracellular ROS levels, as a result, PAH degradation is intrinsically coupled to oxidative stress management [115,119,120].

Detoxification of reactive PAH metabolites is achieved through conjugation reactions mediated by glutathione S-transferases (GST), which increase solubility and enable sequestration or elimination [116,121]. Sustained PAH exposure therefore imposes a high demand on glutathione homeostasis, linking xenobiotic metabolism to glutathione biosynthesis, recycling, and transport. The vacuole plays a central role in this process by sequestering toxic conjugates, while ABC transporters, prevent interaction of reactive intermediates with nuclear and mitochondrial targets [121].

Concomitantly, PAH-tolerant yeasts undergo metabolic reprogramming involving lipid remodeling, engagement of alternative catabolic routes, and redirection of PAH-derived intermediates into central metabolism, while antioxidant systems are induced to limit secondary oxidative damage. Collectively, PAH tolerance in yeasts emerges from systems-level integration of xenobiotic biotransformation, redox balance, vacuolar detoxification, and metabolic flexibility rather than from PAH degradation alone, underpinning both environmental persistence and their value as eukaryotic models of chronic xenobiotic stress adaptation [116,118,121].

#### 2.2.10. Oxidative Stress-Tolerating Yeasts

Tolerance to oxidative stress represents a central feature of eukaryotic extremophily, as reactive oxygen species (ROS) arise as a pervasive consequence of diverse environmental stresses, including salinity, extreme pH, radiation, desiccation, metal exposure, and xenobiotics. Accordingly, oxidative stress–tolerant yeasts have evolved not only to mitigate ROS-mediated damage but also to exploit ROS as regulatory signals coordinating adaptive cellular responses [122].

In yeasts, ROS originate primarily from metabolically active compartments, with mitochondria representing the major source through electron leakage during oxidative phosphorylation, together with contributions from the endoplasmic reticulum during oxidative protein folding and from peroxisomes involved in lipid metabolism [123,124,125]. In addition, the presence of the NADPH oxidase Yno1p indicates that ROS production can be actively regulated as part of cellular signaling networks [126].

ROS play a dual role as signaling molecules and sources of cellular damage. At moderate levels, hydrogen peroxide functions as a second messenger through reversible cysteine oxidation, modulating transcription factors and redox-sensitive enzymes. A canonical example is the Gpx3p–Yap1p pathway, which couples peroxide sensing to transcriptional reprogramming, while mitochondrial cytochrome c peroxidase (Ccp1p) links mitochondrial redox status to nuclear responses [127,128,129]. When ROS levels exceed their signaling range, oxidative damage to DNA, lipids, and proteins activates repair pathways and cell-cycle checkpoints to preserve genomic integrity [130,131,132,133].

Antioxidant defense constitutes the functional core of oxidative stress tolerance in yeasts and involves multiple enzymatic systems, including superoxide dismutases, catalases, glutathione peroxidases, and peroxiredoxins. Beyond detoxification, these enzymes function as redox sensors that integrate oxidative signals with transcriptional and cellular responses. Redox control further depends on glutathione- and thioredoxin-based systems and on NADPH availability as the central reducing currency [127,128,129].

Under oxidative stress, central metabolism is reprogrammed to sustain redox balance, notably through diversion of carbon flux toward the pentose phosphate pathway to enhance NADPH generation, accompanied by induction of genes such as *ZWF1*, *TKL1/2*, and *TAL1.* The accumulation of compatible solutes such as trehalose contributes to stabilization of proteins and membranes under oxidative conditions [134,135,136,137].

At the systems level, oxidative stress tolerance emerges from the integration of redox sensing, signaling, metabolic reprogramming, and cell-cycle control rather than from isolated defense mechanisms. Regulators such as Swi6p modulate growth and global translation, favoring survival-oriented programs over proliferation when oxidative stress is sustained [122,125,138]. This integrative organization positions oxidative stress not as an isolated challenge but as a central hub through which multiple extremophilic traits converge.

### 2.3. Mechanisms of Polyextremotolerance

Yeast-colonized environments are rarely shaped by a single stressor; instead, most natural and anthropogenic niches are polyextreme systems in which multiple physical and chemical pressures coexist. Accordingly, polyextremotolerance should not be interpreted as the simple accumulation of individual stress resistances but as an emergent property arising from the coordinated integration of cellular stress-response networks [13]. Representative environments include solar salterns, hyper-arid deserts and exposed rock surfaces, alkaline lakes, and acid mine drainage systems, where salinity, radiation, pH extremes, nutrient limitation, and heavy metals impose persistent and overlapping selective pressures [13,101].

Across diverse yeast lineages, polyextremotolerance has emerged convergently through the reuse and integration of conserved cellular modules rather than through the evolution of stress-specific pathways. Core regulatory systems, including HOG, CWI, calcineurin, Rim101, TOR, and PKA pathways, interact through extensive crosstalk, enabling context-dependent responses to complex stress combinations (Figure 2) [13,15]. This integration allows yeasts to coordinate osmotic balance, cell wall integrity, ionic homeostasis, and metabolic regulation under conditions where individual stresses cannot be resolved independently [25,48,101].

Redox regulation constitutes a central axis of polyextremotolerance, as most extreme stresses converge on reactive oxygen species accumulation. Polyextremophilic yeasts therefore maintain robust redox-buffering capacity based on glutathione- and thioredoxin-dependent systems, enzymatic antioxidants, and tight control of mitochondrial ROS production [122]. Structural traits such as protective pigmentation further contribute to redox stability by limiting radiation-induced and chemically induced oxidative damage [25,139].

Another defining feature of polyextremotolerance is metabolic organization favoring persistence over rapid growth. In polyextreme environments, where resources are scarce and favorable conditions are transient, yeasts prioritize energy efficiency, cellular maintenance, and survival through metabolic reprogramming and reduced growth rates [15]. This trade-off explains why many polyextremophilic yeasts are poor competitors in nutrient-rich environments yet dominate extreme and fluctuating niches [25,48,54].

From an evolutionary perspective, polyextremotolerance reflects selection in highly variable environments, where adaptive traits initially selected for one stress are reused or exapted to cope with others. This process has driven convergent evolution of regulatory architectures and cellular organization across distantly related fungal lineages, reinforcing the view that polyextremotolerance represents a systems-level property of the eukaryotic cell rather than the outcome of a single genetic or biochemical trait [13,15].

### 2.4. Comparative Advantages Versus Classical Yeasts

Extremophilic yeasts exhibit physiological and organizational features that complement, rather than replace, classical model systems such as *S. cerevisiae*. Whereas *S. cerevisiae* is evolutionarily optimized for rapid growth in stable, nutrient-rich environments, extremophilic yeasts have evolved under conditions characterized by chronic stress, environmental fluctuation, and resource limitation, resulting in enhanced robustness, metabolic flexibility, and capacity for stress integration [3,7,140].

From an experimental perspective, these properties expand the range of biological questions that can be addressed in eukaryotic systems. Extremophilic yeasts are particularly informative for investigating how cells maintain function under sustained chemical and physical stress, how stress-response pathways are rewired under chronic selection, and how trade-offs between growth, maintenance, and survival are resolved at the systems level [2,6]. Extremophilic yeasts enable interrogation of cellular states that are difficult to access in classical laboratory models, where stress responses are often acute, transient, and experimentally imposed.

At the same time, extremophilic yeasts present clear limitations that constrain their experimental deployment, including slow growth rates, strain-to-strain variability, limited genetic toolkits, uneven genomic resources, and reduced experimental controllability under extreme growth conditions. These constraints require careful selection of model species and thoughtful experimental design to balance physiological relevance with tractability. Viewed in this context, extremophilic yeasts should be considered next-generation eukaryotic models in a complementary sense: systems that expand the experimental landscape of yeast biology into regimes of chronic stress, environmental complexity, and metabolic constraint. Their primary conceptual value lies not in demonstrating enhanced tolerance per se, but in revealing alternative organizational principles through which eukaryotic cells integrate stress, reallocate resources, and persist at the limits of viability.

## 3. Current Applications of Yeasts in Scientific Research

Yeast research is increasingly shifting from the use of a single “universal” model organism toward a functional framework in which species are selected according to the biological question and experimental constraints. Within this framework, extremophilic yeasts allow interrogation of physiological, metabolic, and regulatory states that are largely inaccessible in classical models, particularly under chronic, fluctuating, and environmentally relevant stress conditions [6,8].

### 3.1. Classical Uses of Model Yeasts

*S. cerevisiae* has been the principal experimental system for dissecting conserved eukaryotic processes, including cell cycle regulation, DNA replication, intracellular signaling, central metabolism, and protein homeostasis, and it has strongly shaped our mechanistic understanding of eukaryotic cell biology [7,8]. Notably, fundamental pathways such as glycolysis were first systematically characterized in *S. cerevisiae* [141].

The dominance of *S. cerevisiae* as a model organism reflects not only its rapid growth and ease of cultivation but also the early development of powerful genetic toolkits. Approaches such as targeted deletions, controlled gene expression, and reverse genetics enabled the establishment of foundational concepts in genetics, molecular biology, and metabolism, including promoter-dependent transcription, epistasis, homologous recombination, alcoholic fermentation, mitochondrial respiration, and nutrient-dependent metabolic adaptation, primarily under tightly controlled laboratory conditions [7,9,10,11]. Concurrently, *S. cerevisiae* has been central to elucidating conserved processes such as autophagy, the unfolded protein response, vesicular trafficking, and cytoskeletal dynamics, providing mechanistic frameworks later extended to disease-relevant pathways in higher eukaryotes [11].

Classical model yeasts are widely used in metabolic engineering and synthetic biology, where mature genetic and regulatory toolkits support metabolite production, recombinant proteins, and synthetic pathways. However, these systems are typically optimized for performance under controlled, benign, and temporally stable conditions, limiting extrapolation to industrial or environmental settings characterized by chronic stress, fluctuating physicochemical parameters, or sustained chemical complexity [3].

Many processes of biological and biotechnological interest, such as tolerance to chronic oxidative stress, adaptation to hyperosmotic conditions, and prolonged xenobiotic exposure, occur outside the physiological ranges in which *S. cerevisiae* is typically examined, where stress responses are commonly induced transiently under otherwise optimal growth conditions [10,130]. These constraints motivate the use of complementary yeast systems that remain experimentally tractable while better reflecting environmentally realistic regimes characterized by persistent, overlapping stresses.

### 3.2. Emerging Applications Where Extremophilic Yeasts Outperform Classical Models

As research increasingly targets chronic environmental stress, combined stressor exposure, and operational constraints beyond ideal laboratory settings, the limits of classical model systems become more apparent. Addressing these problems requires experimental platforms in which metabolic, regulatory, and structural responses are not only inducible but constitutively organized to function under fluctuating, non-ideal conditions [14,142].

In this context, extremophilic and extremotolerant yeasts are not merely alternatives to classical models but functional experimental systems for studying eukaryotic adaptation under conditions that exceed conventional physiological ranges. By sustaining cellular activity under persistent or combined stresses, these organisms enable analysis of long-term adaptive states and regulatory integration that are difficult to access in classical yeasts optimized for transient stress responses [5,14].

#### Yeasts as Experimental Systems Under Non-Conventional Growth Conditions

Studies of eukaryotic physiology under non-conventional growth conditions highlight extremophilic yeasts as experimentally tractable systems that maintain viability and cellular function in environments that inhibit most microorganisms. Growth at extreme pH, high salinity, or non-standard temperatures enables analysis of cellular robustness, functional stability, and the boundaries of eukaryotic viability under regimes that restrict classical models, while preserving sustained metabolic activity rather than acute stress survival [45,143].

Importantly, the experimental value of extremophilic yeasts lies not only in tolerance but in sustained metabolic activity under stress, enabling analysis of long-term adaptive states rather than transient emergency responses. This provides access to physiological configurations that are rarely observable in standard laboratory models and supports the study of integrated and persistent stress responses within a eukaryotic framework [143,144].

### 3.3. Conceptual Shift: From Universal Models to Functional Model Systems

The growing use of extremophilic yeasts reflects a broader shift in experimental biology, moving beyond a single “universal model organism” toward functional model systems selected for their ecological, physiological, and experimental relevance to specific questions [5,13,45]. This shift does not replace classical models but integrates them: traditional yeasts remain essential for dissecting conserved processes, whereas extremophilic yeasts expose organizational and regulatory features of eukaryotic cells that are difficult to access under standard laboratory conditions [13,145]. From this perspective, the physiological and metabolic diversity of extremophilic yeasts constitutes an experimental strength, enabling comparative analyses of adaptive strategies, cellular resilience, and regulatory plasticity, and supporting a view of the eukaryotic cell as a dynamic system shaped by persistent and interacting environmental pressures [15,26].

### 3.4. Toward a Core Set of Extremophilic Yeast Model Systems

As the diversity of extremophilic and extremotolerant yeasts continues to grow, an important challenge for the field is to move beyond descriptive diversity toward the identification of experimentally robust model systems [13]. Importantly, the value of a yeast as a “model” should not be defined solely by the extent of its extremotolerance, but by practical criteria such as reproducible growth under defined laboratory conditions, availability of genomic resources, experimental tractability, and relevance to well-characterized extreme environments [7].

Across the ecological and physiological contexts discussed in this review, five types of extreme environments emerge as particularly informative for yeast biology: high salinity and ionic stress; low water activity driven by extreme solute concentrations; chronic acidic or alkaline pH; radiation- and desiccation-prone habitats associated with oxidative and genomic damage; and cold or freeze–thaw environments that constrain membrane dynamics and metabolic flux. Together, these settings capture a manageable yet representative range of selective pressures shaping yeast adaptation [15].

Within this framework, a small number of species consistently stand out as promising experimental systems. *D. hansenii* provides a robust model for high-salinity and multi-stress environments, *Z. rouxii* is well suited for studies of extreme osmophily and low water activity, and *W. anomalus* offers a practical balance between broad pH tolerance and experimental accessibility. Among basidiomycetous yeasts, genera such as *Rhodotorula* and *Naganishia* exemplify adaptation to radiation-, desiccation-, and cold-associated stresses, despite more limited genetic toolkits [13,15,16].

Rather than converging on a single universal model organism, the strategic use of a limited set of complementary extremophilic yeasts enables comparative and mechanistic analyses across environmental contexts. This approach supports the integration of physiological and omics data and provides a realistic foundation for identifying conserved and emergent principles of eukaryotic stress adaptation.

## 4. Omics and Systems Biology Advances in Extremophilic Yeasts

**Figure 3:**
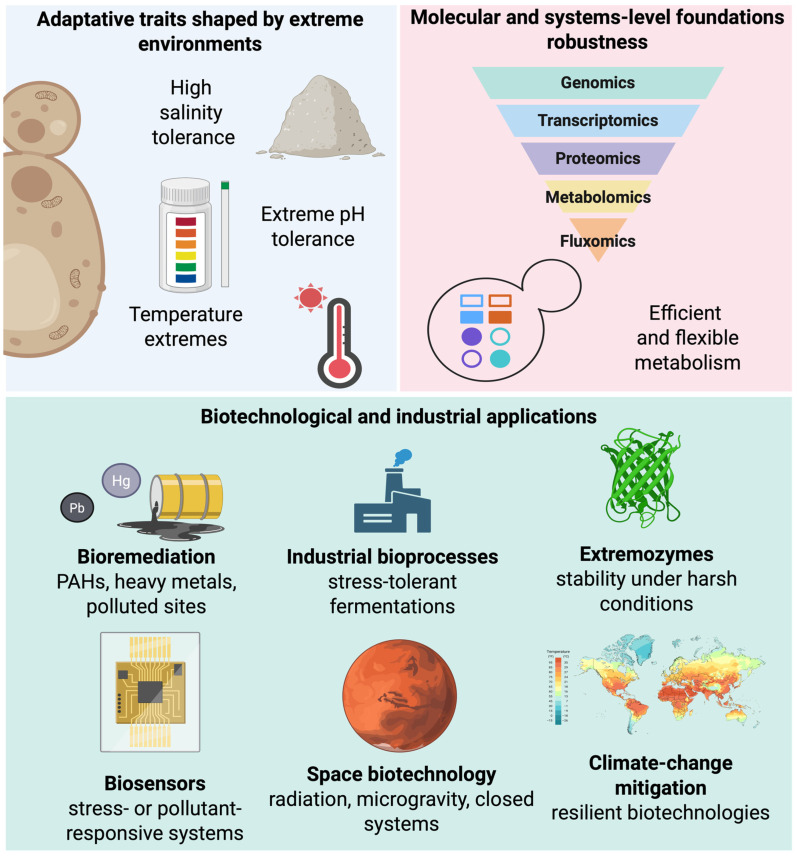
From extremophilic yeasts to applications: a systems-to-technology pipeline. Adaptive traits shaped by extreme environments provide extremophilic yeasts with exceptional cellular robustness. These traits are underpinned by multilayered molecular mechanisms revealed through integrated omics approaches and system-level analyses, including redox control, detoxification networks, and metabolic flexibility. Translating this mechanistic understanding enables the rational development of diverse biotechnological and industrial applications, ranging from bioremediation and stress-tolerant bioprocesses to extremozymes, biosensors, space biotechnology, and climate-change mitigation strategies. Created in BioRender. Padilla, F. (2026) https://BioRender.com/lh96qeo.

Progress in the study of extremophilic yeasts has been driven by omics and systems biology, enabling genomic diversity and adaptive complexity to be integrated into coherent models of cellular function (Figure 3) [146,147]. Unlike classical models, whose genetic and regulatory maps were built over decades under standardized laboratory conditions, extremophilic yeasts are comparatively recent systems in which omics has been essential to capture adaptation under extreme conditions. Integrating genomics, transcriptomics, proteomics, and metabolomics has shifted the field from descriptive extremotolerance toward models that reflect the dynamic nature of cellular stress responses [148,149].

### 4.1. Genomics and Genome Plasticity

Genomic analyses indicate that many extremophilic yeasts possess small- to medium-sized genomes characterized by pronounced plasticity, including gene duplications, expansion of specific gene families, transient aneuploidy, and chromosomal rearrangements. In contrast to the relatively stable genome organization of *S. cerevisiae*, this plasticity is widely considered a key determinant of adaptive flexibility under extreme and fluctuating conditions [150].

In halotolerant yeasts such as *D. hansenii* and *H. werneckii*, genome analyses reveal expansion and redundancy of gene families involved in ion transport, osmotic homeostasis, and stress responses. In *H. werneckii*, extensive genome duplication enables differential regulation of paralogous genes as a function of salinity, illustrating how redundancy supports regulatory rewiring and cellular robustness rather than simple gene dosage effects [44,147,151].

Similarly, in lipogenic yeasts such as *Y. lipolytica*, genomic plasticity is reflected in expanded lipid metabolism and β-oxidation pathways, underpinning metabolic versatility and tolerance to environments enriched in hydrophobic and potentially toxic substrates [152,153].

From an evolutionary perspective, these patterns indicate that adaptation in extremophilic yeasts relies less on isolated genetic innovations than on dynamic genome organization, which enables flexible, system-level responses to persistent environmental pressures [154].

### 4.2. Transcriptomic Responses to Extreme Conditions

Transcriptomic analyses have been central to understanding how extremophilic yeasts integrate multiple environmental signals into coordinated responses. Rather than activating isolated stress programs, they show transcriptional profiles shaped by stress combinations, consistent with the polyextreme nature of their ecological niches [155,156].

In halotolerant and other stress-tolerant yeasts, transcriptomic data reveal coordinated regulation of osmotic signaling, cell wall remodeling, ion transport, and compatible solute metabolism through interconnected regulatory networks, rather than independent pathway activation [28,157]. Similar integrative transcriptional reprogramming has been observed in xenobiotic- and contaminant-tolerant yeasts such as *D. hansenii* and *R. mucilaginosa*, where responses extend beyond detoxification to include central metabolism, redox balance, and energy homeostasis, sustaining viability under chronic chemical stress [116,118].

Importantly, transcriptomic responses to combined stresses in polyextremophilic yeasts are frequently non-additive, generating regulatory states that cannot be predicted from single-stress experiments. This behavior indicates that extremophilic yeasts operate in alternative basal transcriptional regimes, rather than cycling between transient stress-induced responses, underscoring the need for ecologically relevant experimental designs [158].

### 4.3. Proteomics, Metabolomics and Fluxomics

Proteomic and metabolomic analyses provide a functional bridge between transcriptional reprogramming and cellular phenotype in extremophilic yeasts, revealing how stress-induced gene expression translates into reorganization of cellular machinery that sustains viability under extreme conditions [159,160].

At the proteomic level, extremophilic yeasts are enriched in chaperones, antioxidant systems, and detoxification enzymes, many of which undergo post-translational modifications that modulate stability, localization, and activity. These adjustments support protein homeostasis and redox balance under extreme conditions, highlighting a prioritization of maintenance functions over maximal biosynthetic output [161].

Metabolomic analyses further indicate accumulation of compatible solutes, antioxidants, and protective metabolites. In halotolerant species, glycerol and other polyols function as osmoprotectants and redox buffers, while exposure to contaminants induces coordinated rerouting of carbon metabolism toward the pentose phosphate pathway and the glyoxylate cycle, enhancing NADPH generation and stress resilience [32,52,162,163].

Although still limited in scope, fluxomic studies are beginning to show that metabolic fluxes in extremophilic yeasts are redistributed to favor energetic efficiency and redox stability rather than rapid growth. This shift underscores a fundamental trade-off between productivity and persistence, challenging classical growth-centered views of cellular performance under extreme conditions [164].

### 4.4. Synthetic Biology Tools and Genetic Engineering in Non-Conventional Yeasts

The exploitation of extremophilic yeasts in research and biotechnology remains constrained by the limited availability of synthetic biology and genetic engineering tools tailored to their physiology, including efficient transformation systems, well-characterized promoters, and selectable markers [165]. These limitations represent a significant bottleneck for mechanistic studies and currently restrict broader adoption of extremophilic yeasts as standardized model systems.

Recent advances have expanded the application of CRISPR/Cas-based genome editing in non-conventional yeasts, most notably in *Y. lipolytica*, enabling targeted genetic manipulation, metabolic pathway optimization, and development of robust industrial strains [165,166]. Although genetic tools remain comparatively limited in *D. hansenii* and related genera, improved transformation protocols have enabled the generation of targeted mutants, partially alleviating experimental constraints [32]. A persistent challenge, however, is that promoters and regulatory elements effective in *S. cerevisiae* often lose activity under high salinity, extreme pH, or oxidative stress, underscoring the need for context-specific synthetic biology tools adapted to extremophilic conditions (Figure 3) [24,167].

### 4.5. Systems Biology and Integrative Modeling

Integration of omics data has enabled systems biology in extremophilic yeasts, supporting models that capture cellular responses to multiple, concurrent stresses [168]. Unlike classical frameworks that often depict regulatory networks as hierarchical and linear, extremophilic yeasts exhibit highly interconnected networks in which regulatory nodes integrate osmotic, oxidative, thermal, and chemical signals, indicating that extremotolerance emerges from coordinated system-level responses rather than isolated pathways [169,170]. Such modeling provides a realistic framework for eukaryotic adaptation, enabling prediction of cellular behavior and rational design of strains optimized for specific applications (Figure 3) [171].

## 5. Biotechnological and Industrial Applications

Advances in extremophilic yeast physiology have facilitated their translation into applied and industrial settings, particularly in contexts where classical microbial platforms face clear operational limitations. Unlike conventional yeasts optimized for stable, nutrient-rich environments, extremophilic and extremotolerant species maintain metabolic functionality under chronic chemical, osmotic, oxidative, and physicochemical stress, enabling their use in technologically and environmentally demanding applications [145,172].

From a biotechnological perspective, this intrinsic robustness underpins a limited number of application domains in which extremophilic yeasts provide qualitative advantages rather than incremental improvements over classical hosts. In this section, we focus on two areas where their capacity for long-term stress integration is most clearly translated into functional outcomes: (i) environmental technologies, including pollutant transformation, metal detoxification, and bioremediation in contaminated or extreme ecosystems; and (ii) industrial bioprocesses designed to operate under hostile conditions with reduced sterility requirements, lower contamination risk, and enhanced operational stability. While these applications highlight the potential of extremophilic yeasts, their broader industrial deployment remains constrained by factors such as growth rates, genetic tool availability, and process scalability, which are discussed below (Figure 3) [16,145].

### 5.1. Bioremediation and Biodegradation of Environmental Pollutants

The bioremediation of persistent environmental contaminants represents one of the most mature and impactful application areas for extremophilic yeasts. Their ability to maintain metabolic and regulatory activity under chronic chemical toxicity, oxidative stress, extreme pH, high salinity, and nutrient limitation enables contaminant transformation under conditions that commonly co-occur at polluted sites, where conventional microbial systems are severely constrained [115].

Importantly, the biotechnological value of extremophilic yeasts in bioremediation lies not merely in tolerance to toxic compounds, but in their capacity for sustained detoxification under in situ conditions. Their eukaryotic cellular organization supports integration of detoxification, redox control, and metabolic reprogramming, enabling long-term functionality and persistence in complex environments where many prokaryotic platforms exhibit rapid loss of activity [15,142].

#### 5.1.1. Degradation of Hydrocarbons and PAHs

Extremophilic and non-conventional yeasts display strong potential for the degradation and transformation of aliphatic hydrocarbons and PAHs, including highly recalcitrant compounds such as benzo(a)pyrene. Genera such as *Candida*, *Debaryomyces*, *Rhodotorula*, and *Yarrowia* maintain metabolic activity under adverse conditions, including high salinity, nutrient limitation, and chronic chemical stress, supporting their suitability for in situ bioremediation strategies in contaminated environments [115].

From an applied perspective, PAH degradation by yeasts depends on an integrated cellular phenotype that supports sustained detoxification during prolonged exposure rather than on isolated catabolic reactions. Eukaryotic compartmentalization enables effective management of reactive intermediates, mitigation of membrane perturbation and oxidative burden, and metabolic integration of PAH-derived products. As a result, extremophilic yeasts prioritize long-term viability and functional persistence over rapid growth, conferring a qualitative advantage in polyextreme environments where resilience and sustained activity are critical determinants of bioremediation success and where bacterial systems frequently lose functionality [116,118,121].

#### 5.1.2. Metal Tolerance and Heavy Metal Remediation

Heavy metals represent persistent environmental contaminants that impose chronic toxicity through disruption of redox balance, membrane integrity, protein function, and metal homeostasis. Because metals are not degradable, effective bioremediation relies on cellular tolerance and controlled immobilization rather than elimination. Under these conditions, extremophilic yeasts function as robust eukaryotic platforms capable of sustaining metabolic and regulatory activity in metal-contaminated environments where conventional microorganisms exhibit limited performance [113,142].

Numerous extremophilic and extremotolerant yeasts display resistance to metals such as Cd, Pb, Zn, Cu, and Cr, supporting their application in biosorption, bioaccumulation, and detoxification-based remediation strategies. Genera including *Debaryomyces*, *Rhodotorula*, *Yarrowia*, and *Candida* combine metal tolerance with resilience to additional stresses such as salinity, extreme pH, and oxidative pressure, a combination particularly advantageous in polyextreme contaminated sites [142]. Their effectiveness derives from the capacity to maintain cellular viability and regulatory integration under chronic metal exposure rather than from single resistance mechanisms.

At the mechanistic level, metal remediation by yeasts emerges from the integration of extracellular binding, intracellular chelation, vacuolar sequestration, and redox buffering, collectively limiting metal reactivity and cytotoxicity. In some species, additional transformation processes, such as the reduction in Cr(VI) to the less toxic Cr(III), further enhance remediation efficiency by coupling cellular detoxification with environmentally relevant chemical stabilization [113,142]. Together, these features position extremophilic yeasts as versatile and resilient platforms for heavy metal remediation in complex environments, where long-term functionality and stress integration are essential.

#### 5.1.3. Co-Metabolic Systems and Microbial Consortia

In complex contaminated environments, effective pollutant removal often depends on the coordinated activity of microbial consortia, in which different organisms perform complementary steps of transformation, detoxification, or assimilation. Within these systems, extremophilic yeasts frequently play strategic roles that extend beyond direct contaminant degradation, acting as stabilizing and facilitating partners under severe chemical and physicochemical stress [173].

By tolerating salinity, extreme pH, metal toxicity, and oxidative stress, extremophilic yeasts reduce local toxicity and sustain metabolic activity in conditions that inhibit many bacterial degraders. Through detoxification, redox buffering, and compartmentalization of toxic intermediates, they generate microenvironments that support the persistence and activity of more sensitive microbial partners involved in downstream degradation processes [174].

This functional complementarity is particularly relevant in polyextreme systems, where remediation efficiency emerges from synergistic interactions rather than from the performance of individual species. In this context, extremophilic yeasts contribute to consortium robustness and long-term functionality by maintaining metabolic continuity under fluctuating and hostile conditions, reinforcing their value as key components of engineered and natural bioremediation consortia [175].

### 5.2. Industrial Production Platforms

Extremophilic yeasts have emerged as attractive industrial production platforms due to their capacity to sustain metabolic activity under physicochemical conditions that inhibit contaminant proliferation and constrain the performance of conventional microbial hosts. Their tolerance to extremes of pH, salinity, temperature, and chemical stress enables bioprocesses that operate beyond the physiological limits of classical yeasts, enhancing operational robustness and long-term process stability [176].

Crucially, this robustness supports sustained production under chronic and fluctuating conditions, shifting the industrial paradigm from tightly controlled, sterile environments toward processes compatible with variable substrates and stress-prone operating regimes. In this context, extremophilic yeasts are best viewed not simply as alternative production hosts, but as platforms whose intrinsic stress integration aligns with realistic industrial constraints and sustainability-driven process design [16,145].

#### 5.2.1. Biofuels and Lipid-Based Products

The production of biofuels and lipid-derived compounds represents a major application area in which non-conventional and extremotolerant yeasts offer clear operational advantages. Among these, *Y. lipolytica* has emerged as a reference industrial chassis due to its strong lipogenic capacity and efficient assimilation of hydrophobic substrates, including waste oils, fatty acids, and industrial by-products [177].

*Y. lipolytica* combines lipid accumulation with tolerance to osmotic, chemical, and nutritional stress, enabling sustained metabolic activity under non-ideal and fluctuating process conditions. This robustness supports stable lipid biosynthesis while reducing sensitivity to feedstock variability and operational perturbations [31,178].

From an applied perspective, the intrinsic stress tolerance of lipogenic yeasts facilitates flexible fermentation strategies with reduced dependence on strict environmental control. Metabolic engineering has further expanded their industrial relevance by redirecting carbon fluxes toward tailored fatty acids, surfactants, and high value–added lipid-based products, reinforcing their suitability for large-scale biofuel and oleochemical production [31,177].

#### 5.2.2. Organic Acids, Enzymes and Specialty Chemicals

Extremophilic and extremotolerant yeasts are increasingly exploited for the production of organic acids, industrial enzymes, and specialty chemicals, particularly in bioprocesses operating under extreme physicochemical conditions. Acidophilic and alkalitolerant yeasts can sustain metabolite production at pH ranges that inhibit most contaminants, simplifying downstream processing and reducing sterility requirements [179,180].

Beyond contamination control, operation under extreme pH, salinity, or temperature enables production systems that preserve catalytic activity and product stability under conditions that compromise classical hosts. In this context, extremophilic yeasts provide a functional advantage by coupling stress tolerance with eukaryotic traits such as efficient secretion, protein folding, and post-translational modification. These properties underpin the growing use of yeast-derived extremozymes in food processing, detergents, biocatalysis, and environmental applications requiring long-term operational stability [13,179,181].

#### 5.2.3. Process Robustness, Sustainability and Future Industrial Perspectives

The deployment of extremophilic yeasts in industrial biotechnology enables bioprocesses that operate under realistic industrial and environmental constraints. Their capacity to sustain metabolic activity under extreme physicochemical conditions reduces contamination risk, relaxes operational control requirements, and supports the use of non-conventional substrates and waste streams, lowering energy input and overall process complexity [16,144].

Beyond operational advantages, these properties align extremophilic yeasts with sustainability-driven process design. Their robustness facilitates circular and resource-efficient bioprocesses by enabling the valorization of chemically challenging residues and variable feedstocks, integrating economic feasibility with environmental responsibility [16,145].

#### 5.2.4. Non-Sterile and Low-Cost Bioprocesses

Extremophilic yeasts enable industrial bioprocesses under non-sterile or reduced-sterility conditions by operating at pH, salinity, or temperature ranges that suppress the growth of most contaminating microorganisms. This intrinsic selectivity simplifies process design, reduces energy and infrastructure demands associated with sterilization, and improves economic feasibility at scale [18,142,182].

Such characteristics are particularly advantageous for decentralized, low-cost, or resource-limited applications involving waste streams or variable feedstocks, where strict environmental control is impractical. In these settings, extremophilic yeasts provide a pragmatic balance between biological performance and process robustness.

### 5.3. Yeast-Based Biosensors

Extremophilic and extremotolerant yeasts provide robust platforms for whole-cell biosensors designed to operate under harsh and fluctuating environmental conditions. Their ability to maintain viability, regulated gene expression, and signal transduction under extreme physicochemical stress enables reliable sensing in environments where conventional microbial biosensors rapidly lose functionality [142].

Yeast-based biosensors integrate sensing, signal processing, and stress tolerance within a single eukaryotic chassis. In extremophilic yeasts, this integration is particularly advantageous because stress-response pathways are constitutively embedded in cellular organization rather than transiently induced, allowing stable signal output during prolonged exposure to metals, xenobiotics, or oxidative stress-inducing compounds [183].

Compared with bacterial systems, yeasts offer enhanced genetic stability, compartmentalization, and regulatory control, supporting long-term deployment under variable conditions. These features position extremophilic yeasts as promising living sensors for in situ environmental monitoring, industrial process control, and long-term surveillance in polyextreme or contaminated settings where robustness and signal persistence are critical [184].

### 5.4. Conceptual Implications for Sustainable Biotechnology

Beyond their established roles in bioremediation and industrial production, extremophilic yeasts provide a conceptual framework for sustainable biotechnology grounded in robustness rather than maximal productivity. Their capacity to integrate stress tolerance, metabolic plasticity, and regulatory control enables biological processes to operate under harsh, fluctuating, and resource-limited conditions that more closely reflect real-world industrial and environmental constraints, supporting long-term functionality beyond the limits of classical microbial platforms [13,144,145].

## 6. Challenges and Knowledge Gaps

Despite a growing body of research and accumulating evidence supporting extremophilic yeasts as eukaryotic models and biotechnological platforms, structural and conceptual limitations still restrict their broader adoption. These constraints highlight an incomplete shift from paradigms centered on classical model organisms toward functional, context-driven approaches. Addressing them is essential to consolidate extremophilic yeasts as reliable experimental systems and applied biotechnological tools [13,18].

A major obstacle in studying extremophilic yeasts is the limited availability of standardized genetic tools. Unlike *S. cerevisiae*, which benefits from well-established toolkits, many extremophilic yeasts lack robust, reproducible systems for genetic manipulation, restricting experimental accessibility, functional validation, and cross-study comparability [143].

Although more genomes have been sequenced, functional annotation in extremophilic yeasts remains incomplete, with many genes lacking clear homologs or containing poorly characterized domains, particularly those linked to extreme adaptation. This limitation hampers mechanistic interpretation of omics datasets and constrains the development of predictive, system-level models [146,185]. Melanized black fungi and polyextremophilic yeasts exemplify this challenge, as their genomes often display extensive duplications and structural rearrangements that complicate functional inference and exceed the resolution of conventional annotation frameworks [95,147].

At the translational level, converting laboratory insights into industrial applications remains challenging. Many extremophilic yeasts grow more slowly than classical models, limiting volumetric productivity in large-scale bioprocesses [142,186]. In addition, these organisms often display specific nutritional demands and non-linear physiological responses to changes in salinity, pH, or oxygen availability, complicating process design, control, and optimization, particularly in the absence of well-defined kinetic and physiological parameters [14,187]. The scarcity of systematic scale-up studies and comparative frameworks with classical yeasts has reinforced a perception of technological risk, which continues to limit industrial adoption at scale [186].

Although omics approaches have generated extensive datasets on extremophilic yeasts, their integration into mechanistic and predictive frameworks remains limited, as most studies focus on individual omics layers and capture only partial aspects of cellular responses [146]. In polyextremophilic yeasts, multiple stresses interact non-additively, generating emergent behaviors that cannot be resolved through single-layer analyses. Without standardized strategies for multi-omics integration, cross-study comparability is restricted and extrapolation to applied contexts remains constrained [185].

Overcoming these limitations will require integrative pipelines and quantitative approaches capable of capturing the dynamic behavior of highly interconnected cellular systems [146]. Equally important is the development of standardized methodologies to evaluate the biotechnological performance of extremophilic yeasts, as current studies often rely on heterogeneous experimental conditions that limit reproducibility and hinder direct comparison. This need spans from defining operationally meaningful “extreme conditions” to harmonizing functional assays assessing growth, viability, enzymatic activity, and contaminant degradation. Developing such comparable protocols will be essential to consolidate the field and support systematic advancement [14,148].

## 7. Future Perspectives

Despite the challenges outlined above, extremophilic yeasts are increasingly relevant to eukaryotic biology and future biotechnological innovation. Their physiological diversity and adaptive robustness offer a strong framework to meet emerging scientific and technological demands [188].

Extremophilic yeasts are emerging as next-generation eukaryotic systems for synthetic biology and applied biotechnology, as their physiological robustness supports processes that remain functional under hostile conditions, reducing dependence on tightly controlled environments [16]. As genetic tools mature, integration with metabolic engineering and synthetic regulatory approaches is expected to shift bioprocess design from strict environmental control toward biologically embedded robustness [14].

Climate change is intensifying extreme environmental conditions, including droughts, soil salinization, ocean acidification, and the accumulation of persistent contaminants, positioning extremophilic yeasts as relevant contributors to mitigation and adaptation strategies [5]. Their capacity to integrate stress tolerance with metabolic functionality under adverse conditions reinforces their relevance as biological tools for emerging environmental challenges [189].

The integration of multi-omics data with artificial intelligence and machine learning approaches offers a powerful framework to uncover hidden patterns in large datasets, predict cellular responses to combined stresses, and support the rational design of optimized strains. Such integrative approaches are particularly well suited to extremophilic yeasts, in which stress responses are non-additive and emerge from system-level network reorganization rather than isolated pathways. Predictive models grounded in systems biology can thereby accelerate the development of extremophilic yeasts tailored to specific applications while reducing experimental time and costs [185,190].

Extreme space conditions, including ionizing radiation, microgravity, vacuum, and limited resources, align with the stress tolerance profiles of extremophilic yeasts, supporting their use as models for astrobiology and for the development of biological life-support systems. Here again, their value lies not in single tolerance traits, but in the integration of stress resistance, metabolic stability, and long-term functionality under resource-limited conditions, enabling biomolecule production, waste recycling, and bioremediation beyond Earth [89,191].

## 8. Conclusions

Extremophilic yeasts represent an important eukaryotic model for scientific research. For practical purposes, a limited set of reference systems can already be identified, including *D. hansenii* for salinity stress, *Z. rouxii* for extreme osmotic conditions, *W. anomalus* for pH extremes, *Rhodotorula* species for cold adaptation, and *Naganishia* species for radiation and desiccation tolerance. Together, these organisms span a broad yet tractable range of environmental pressures that shape eukaryotic life under extreme conditions.

Their taxonomic diversity, physiological plasticity, and ability to integrate responses to multiple stresses position them as complementary systems to classical models under environmentally relevant conditions. Importantly, beyond extending tolerance limits, these yeasts reveal a mode of eukaryotic organization in which stress integration is a constitutive feature of cellular physiology rather than a transient, inducible response. This organizational principle provides conceptual insights into eukaryotic robustness, network-level coordination, and metabolic trade-offs that are not readily accessible in conventional laboratory models.

Beyond their value as experimental systems, extremophilic yeasts constitute versatile biotechnological platforms with significant potential to support environmental sustainability, industrial innovation, and emerging applications such as space biotechnology. Progress in the field will depend on the consolidation of genetic and synthetic biology tools, methodological standardization, and the integration of multi-omics and computational approaches. While these challenges remain non-trivial, the trajectory toward their adoption as next-generation eukaryotic models is already clearly defined.

Importantly, future progress in the field will depend less on expanding the catalog of extremophilic yeasts and more on consolidating a limited number of experimentally robust model systems. Focusing on such models will facilitate mechanistic dissection of stress integration, enable meaningful cross-study comparisons, and accelerate the translation of omics-based insights into biotechnological and biosensing applications.

## Figures and Tables

**Table 1:** Representative yeast species exhibiting extremophilic and extremotolerant traits.

Extremophilic Trait	Fungal Group	Representative Species
Thermophilic/Thermotolerant	Ascomycota	*Blastobotrys adeninivorans*, *Kluyveromyces marxianus*, *Lachancea thermotolerans*, *Ogataea polymorpha*, *Sporopachydermia lactativora*
	Basidiomycota	*Cryptococcus neoformans*, *Cystobasidium benthicum*
Psychrophilic/Psychrotolerant	Ascomycota	*Candida psychrophila*, *Metschnikowia australis*, *Taphrina antarctica*
	Basidiomycota	*Glaciozyma watsonii*, *Mrakia gelida*, *Naganishia vishniacii*, *Vishniacozyma carnescens*
Halophilic/Halotolerant	Ascomycota	*Debaryomyces subglobosus*, *Metschnikowia bicuspidata*, *Schwanniomyces etchellsii*
	Basidiomycota	*Naganishia albida*, *Rhodotorula sphaerocarpa*, *Sterigmatomyces halophilus*
	Black yeasts	*Hortaea werneckii*, *Wallemia ichthyophaga*
Osmophilic/Osmotolerant	Ascomycota	*Zygosaccharomyces mellis*, *Candida zemplinina*
Xerotolerant	Ascomycota	*Candida versatilis*, *Zygosaccharomyces bisporus*
Acidophilic/Acidotolerant	Ascomycota	*Kazachstania exigua*, *Cyberlindnera saturnus*
	Basidiomycota	*Rhodotorula toruloides*, *Tremella fuciformis*
Alkali-tolerant	Ascomycota	*Komagataella pastoris*, *Metschnikowia pulcherrima*, *Wickerhamia fluorescens*
	Black yeasts	*Exophiala alcalophila*

**Table 2:** Polyextremophilic and extremotolerant yeast species.

Species	Fungal Group	Extremophilic Traits *
*Debaryomyces hansenii*	Ascomycota	A, AL, H, P, X
*Naganishia adeliensis*	Basidiomycota	A, AL, H, P, X
*Zygosaccharomyces rouxii*	Ascomycota	A, H, O, X
*Aureobasidium pullulans*	Black yeasts	AL, H, P

* A = acidophilic/acidotolerant; AL = alkali-tolerant; H = halophilic/halotolerant; O = osmophilic/osmotolerant; X = xerotolerant; P = psychrophilic/psychrotolerant.

## Data Availability

No new data were created or analyzed in this study. Data sharing is not applicable to this article.
